# NGO ameliorates psoriasis by modulating mitochondrial function and suppressing pSTAT3–IL-17–expressing CD8^+^ TRM cells

**DOI:** 10.1186/s12951-025-04020-7

**Published:** 2026-01-16

**Authors:** Tae Ho Kim, Chae Rim Lee, Kyoung Min Choi, Soon Won Choi, Seon-Yeong Lee, A. Ram Lee, Jung Won Choi, Young Mi Moon, Joonhyuck Park, Haeyoun Choi, Jaechul Ryu, Chul Hwan Bang, Mi-La Cho

**Affiliations:** 1 Lab of Translational ImmunoMedicine (LaTIM), Catholic Research Institute of Medical Science, College of Medicine, The Catholic University of Korea, 222, Banpo-daero, Seocho-gu, Seoul, 06591 Republic of Korea; 2 Department of Pathology, College of Medicine, The Catholic University of Korea, Seoul, 06591 South Korea; 3 Department of Biomedicine & Health Sciences, College of Medicine, The Catholic University of Korea, Seoul, 06591 Korea; 4 Department of Dermatology, College of Medicine, Seoul St. Mary’s Hospital, The Catholic University of Korea, 222, Banpo-daero, Seocho-gu, Seoul, 06591 Republic of Korea; 5 Institutes of Convergence Technology, INBCT Co.LTD, Seoul, 08826 Republic of Korea; 6 Department of Microbiology, The Catholic University of Korea, Seoul, 06591 South Korea; 7 Department of Medical Life Science, College of Medicine, The Catholic University of Korea, 222, Banpo-daero, Seocho-gu, Seoul, 06591 Republic of Korea

**Keywords:** Psoriasis, NGO, Mitochondria, STAT3, CD8^+^ TRM cell, IL-17

## Abstract

Psoriasis is a chronic autoimmune skin disorder characterized by keratinocyte hyperproliferation, persistent inflammation, and frequent relapse. Tissue-resident memory T (TRM) cells, particularly IL-17– and phosphorylated STAT3 (pSTAT3)–expressing CD8^+^ subsets, have been established as central drivers of disease persistence and recurrence. These pathogenic TRM cells maintain local inflammatory circuits that are poorly targeted by current therapies, which helps to explain the limited durability of clinical remission in many patients. Therefore, strategies for selective suppression of these immune subsets are urgently needed. In this study, we investigated the therapeutic potential of nano-sized graphene oxide (NGO), a biocompatible material with emerging anti-inflammatory properties. NGO treatment significantly enhanced mitochondrial function in immune cells, reflected by improved oxygen consumption rate and reduced mitochondrial reactive oxygen species production. Restoration of mitochondrial homeostasis led to robust suppression of pSTAT3 and IL-17 signaling pathways. Importantly, NGO induced a dose-dependent reduction in the numbers of pathogenic CD8^+^ TRM17 cells in both murine models of psoriasis and human-patient-derived immune cells. Beyond its direct effects on pathogenic TRM cells, NGO treatment alleviated epidermal hyperplasia and inflammatory infiltration in psoriatic lesions and promoted the expansion of regulatory T cells, thereby fostering an immune environment more conducive to tissue resolution. Taken together, these findings show that NGO exerted potent therapeutic effects by coupling mitochondrial reprogramming with immune modulation. By attenuating pSTAT3–IL-17–driven TRM cell responses and enhancing regulatory T cell activity, NGO has emerged as a promising candidate for durable immunometabolic therapy in psoriasis.

**Graphical abstract:**

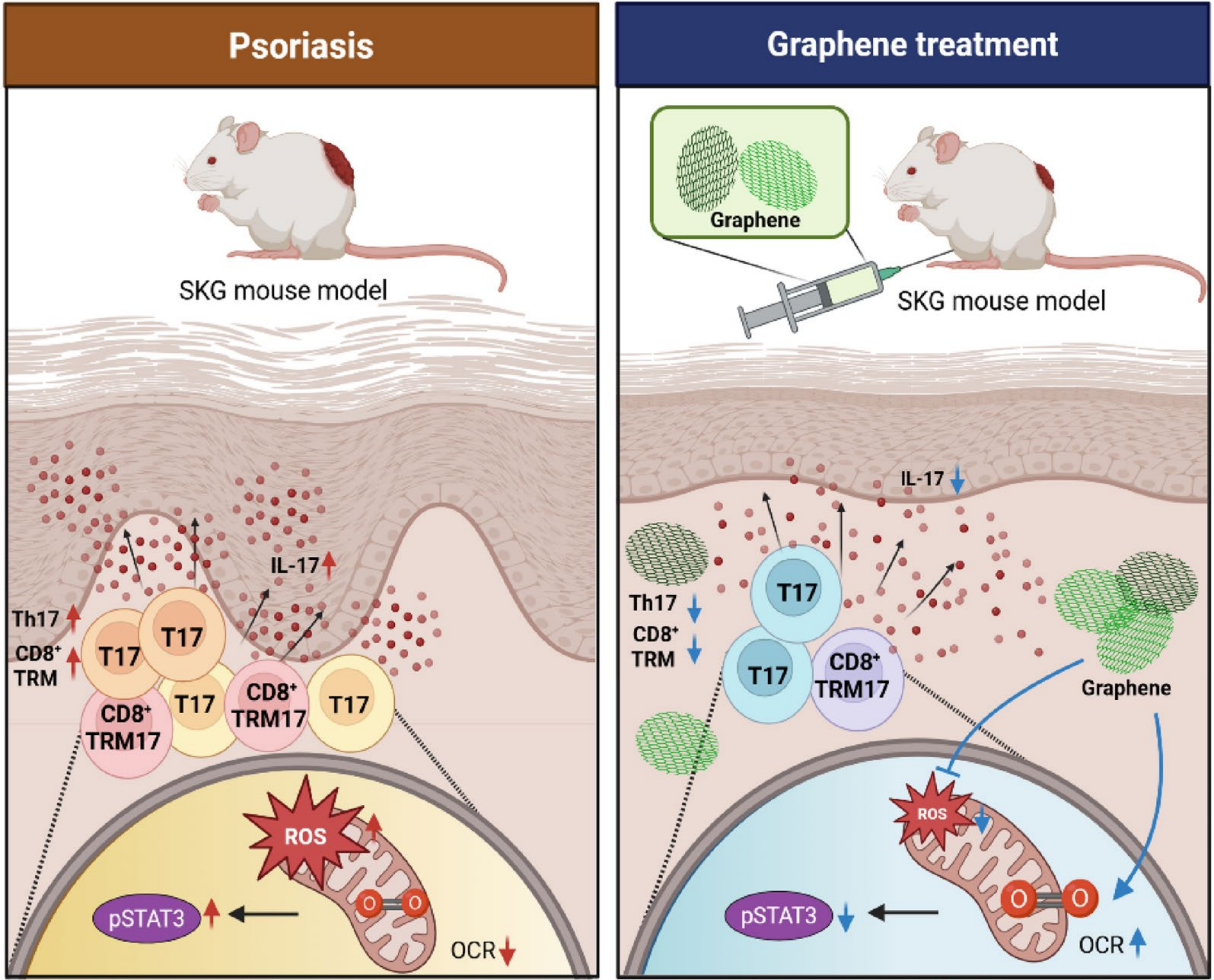

**Supplementary Information:**

The online version contains supplementary material available at 10.1186/s12951-025-04020-7.

## Introduction

Psoriasis is a prevalent, chronic, immune-mediated inflammatory skin disorder that affects more than 60 million individuals worldwide. It is characterized by keratinocyte hyperproliferation and infiltration of immune cells, leading to erythematous and scaly plaques [1]. Beyond the cutaneous involvement, psoriasis is frequently associated with a number of comorbidities such as psoriatic arthritis, cardiometabolic diseases, and psychiatric disorders, which substantially impair the quality of life of patients [1, 2]. Despite significant advances in therapeutic strategies, including phototherapy, conventional systemic agents, and biologics, many patients continue to experience limited efficacy, adverse effects, or disease relapse, highlighting the urgent need for novel treatment approaches [2]. Recent work has implicated tissue “inflammatory memory,” including epigenetic reprogramming, noncoding RNA/protein network regulation, and tissue remodeling driven by tissue-resident memory T (TRM) cells, in disease persistence and recurrence [3].

Skin TRM cells are a distinct, long-lived, non-recirculating subset of memory T lymphocytes that provide rapid, localized defense at barrier surfaces [4]. Dysregulation of TRM cells has been implicated in multiple autoimmune and inflammatory diseases, including vitiligo, chronic eczema, asthma, inflammatory bowel disease, autoimmune hepatitis, and psoriasis [5]. In psoriasis, CD69^+^CD103^+^CD8^+^ TRM cells are enriched in both lesional and clinically nonlesional skin and have a heightened capacity for IL-17 A production compared with healthy skin [6–8]. These TRM cells also express CCR6, IL-23R, and persist long-term in clinically healed psoriasis lesions. Upon stimulation, CD8^+^ TRM cells produce IL-17 A, while CD4^+^ T cells produce IL-22, contributing to localized inflammation and the potential for disease relapse [9]. Consequently, these cells, together with the IL-23/STAT3–IL-17 signaling axis, play a central role in initiating and sustaining chronic cutaneous inflammation. Although calcipotriol/betamethasone dipropionate and anti-IL-23 monoclonal antibodies have been reported to reduce total and IL-17–producing TRM cell numbers, the evidence remains limited and these interventions are not curative, highlighting the need for strategies to address directly TRM cell persistence and pathogenic programs [10–12].

Recent studies further indicate that mitochondrial reactive oxygen species (mtROS) act as key upstream modulators of STAT3 phosphorylation [13, 14]. Elevated mtROS promotes STAT3 activation, which in turn drives IL-17 gene transcription and sustains the inflammatory functions of pathogenic TRM cells. Thus, excessive mtROS contribute to the persistence of STAT3-dependent inflammatory loops in psoriasis, whereas reduction of mtROS may represent an effective means to attenuate STAT3 activity and IL-17 production [15, 16]. Accordingly, attenuating STAT3 activation by reducing mitochondrial ROS is crucial for limiting IL-17 secretion from CD8^+^ TRM cells, thereby alleviating pathogenic inflammatory responses in psoriasis.

In response to these issues, nano-sized graphene oxide (NGO) is being explored as a versatile cutaneous drug-delivery platform. Its large specific surface area, abundant oxygen-containing groups, and facile surface functionalization enable high-capacity loading and targeted delivery of small molecules, anti-inflammatory biologics, or nucleic acids to inflamed skin [17]. In addition to acting as a carrier, preclinical studies have suggested that NGO has intrinsic antioxidant and anti-inflammatory activities capable of dampening reactive oxygen species (ROS)–driven and cytokine-mediated pathways [18], implying that NGO-based formulations may localize and potentiate antipsoriatic therapies within TRM cell-enriched lesional microenvironments of psoriasis. In addition, the biocompatibility profile of NGO is favorable, with relatively low cytotoxicity at controlled doses, supporting its topical or transdermal use [19, 20]. There is accumulating evidence that NGO can suppress keratinocyte hyperproliferation and modulate cutaneous immune cell activity [21, 22], pathways that are central to chronic inflammatory dermatoses such as psoriasis. Based on these properties, NGO is an attractive, multifunctional platform for next-generation skin-targeted therapies that may help to overcome the efficacy, safety, and durability limitations of current treatments.

Here, we examined whether NGO can modulate mtROS-STAT3-IL-17 axis in TRM cell programs and attenuate keratinocyte hyperproliferation, thereby enhancing antipsoriatic efficacy in preclinical models.

## Materials and methods

### Animals

Male SKG mice were maintained in a specific-pathogen-free environment. All protocols involved in the animal experiments were reviewed and approved by the Institutional Animal Care and Use Committee (IACUC) of the Department of Laboratory Animals at the College of Medicine, Catholic University of Korea (Permit Number: 2023-0110-04). All procedures were conducted in accordance with the guidelines of the National Institutes of Health (NIH).

### Psoriasis induction and treatment

Psoriasis was induced in 10-week-old male SKG mice by daily topical application of 5% imiquimod (IMQ) cream (62.5 mg per mouse, NDC 45802-368-62; Dong-A ST, Seoul, South Korea) to the shaved dorsal skin for 6 consecutive days. Mice were sacrificed on day 7 for analysis. To assess its therapeutic effect, NGO (31NM-0008; INBCT Co., Ltd., Seoul, South Korea) was administered intraperitoneally at a dose of 6.7 mg/kg three times per week, beginning concurrently with application of IMQ. Two experimental groups were established: an acute psoriasis model (SKG + IMQ, *n* = 6) and an NGO treatment group (NGO, *n* = 8).

### Preparation and characterization of NGO

NGO preparations, obtained from INBCT Co., Ltd., were suspended in deionized water. NGO was synthesized from graphite via Taylor-Couette flow. The morphology of the synthesized NGO particles was analyzed using Cs-corrected high-resolution transmission electron microscopy (HRTEM) (JEM-ARM200F, Cold FEG; JEOL Ltd., Tokyo, Japan) after the material had been loaded onto a 400-mesh carbon-coated copper grid. The surface charge of the sample was determined using a Zetasizer Nano ZS (Malvern Instruments, Malvern, Worcestershire, UK) equipped with a clear disposable zeta cell. Samples were dispersed in deionized water (dispersant refractive index 1.330, dielectric constant 78.5, viscosity 0.8872 cP) at 25 °C. The size distribution of the NGO was determined using a disc stack centrifuge (CPS Instruments, Prairieville, LA, USA). In addition, NGO samples prepared on sapphire wafers were examined by atomic force microscopy (AFM) in noncontact mode (scanned area: 25 µm^2^, XE-100; Park Systems, Suwon, South Korea).

### Cell viability assay

Cell viability was determined using a Cell Counting Kit-8 assay (CCK-8; Dojindo Laboratories, Kumamoto, Japan), which is based on the reduction of WST-8 by mitochondrial dehydrogenases in viable cells. Immortalized human keratinocytes (HaCaT) were seeded in 96-well plates at a density of 5 × 10^3^ cells/well and allowed to attach overnight. Cells were then treated with NGO at final concentrations of 3.13, 6.25, 12.5, 25, and 50 µg/ml for 24 h under standard culture conditions (37 °C, 5% CO_2_). Following treatment, 10 µL of CCK-8 solution was added to each well containing 100 µL of culture medium and incubated for 2 h. The absorbance was measured at 450 nm using a microplate reader (SpectraMax; Molecular Devices, San Jose, CA, USA). Cell viability was expressed as a percentage relative to untreated control cells.

### Clinical assessment of psoriasis

The clinical severity of psoriasis in the mouse model was assessed using criteria analogous to those used in human patients. Key clinical features (i.e., erythema, scaling, and skin thickness) were evaluated using a modified Psoriasis Area and Severity Index (PASI) scoring system, with values ranging from 0 to 4. Specifically, a score of 0 indicated no psoriatic features; 1 corresponded to mild erythema and dorsal skin thickness of approximately 1 mm; 2 represented moderate redness, distinct ridging, thickness of 1–2 mm, and scaling along the creases; 3 denoted moderately dark redness, diffuse ridging, thickness > 2 mm, and moderate scaling over a wider area; and 4 indicated severe dark redness, tightly ridged skin, and extensive scaling. PASI scores for each parameter were determined by direct visual inspection, and all measurements were obtained independently by two investigators.

### Histopathological analysis

Dorsal skin samples were excised carefully following sacrifice of the acute psoriasis model mice after 1 week (day 7). The tissues were fixed in 10% neutral-buffered formalin for 48 h and processed for paraffin embedding. Paraffin-embedded tissue blocks were sectioned at a thickness of 5 μm using a rotary microtome (Leica Biosystems, Seoul, South Korea). Standard hematoxylin (S2-5; Youngdong Pharmaceutical Co., Ltd., Seoul, South Korea) and eosin (32002; Muto Pure Chemicals Co., Ltd., Tokyo, Japan) staining (H&E) was performed. For histological assessment, three representative fields per slide were selected, and the epidermal thickness was measured at six distinct points per field. The mean values from these measurements were used to define the histological score.

### Phenotypic profiling of tissue-resident memory CD8^+^ T cells

Paraffin-embedded skin and spleen Sect. (5 μm thick) and cytospin preparations of isolated splenocytes and patient-derived PBMCs were prepared for immunofluorescence staining. To identify skin-resident memory CD8^+^ T cells, sections were incubated with primary antibodies against the surface markers CD8 (NBP1-49045; Novus Biologicals, Littleton, CO, USA) and CD103 (AF1990; R&D Systems, Minneapolis, MN, USA). To detect key functional proteins, additional staining was performed using anti-CD69 (PA5-102562; Invitrogen, Carlsbad, CA, USA), anti-IL-17 (ab79056; Abcam, Cambridge, MA, USA), and anti-phospho-STAT3 (pSTAT3) (Y705, ab76315) antibodies. Nuclear counterstaining was performed with DAPI (D3571; Invitrogen). Stained samples were visualized with a confocal laser scanning microscope (LSM700 and LSM900 w/Airyscan II; Carl Zeiss, Oberkochen, Germany). Quantification of marker expression and analysis of double-positive cells at the dermal–epidermal junction were performed by manual cell counting using ZEISS ZEN microscopy software. Immunofluorescence images were acquired at 200× magnification, with approximately three representative fields per slide selected for analysis. Quantification was achieved by averaging the number of target-positive cells across these fields, analyzing on average 140 to 170 DAPI-stained cells per field in cell confocal analysis. Analysis in skin tissues focused on psoriatic lesion regions with epidermal thickening and immune cell infiltration, while splenic tissue evaluation targeted immune cell clusters. Positive cell counts were based on co-localization of FITC, PE, APC, and DAPI signals, ensuring consistent and reproducible measurement of marker expression.

### Immunohistochemistry

Paraffin-embedded skin tissues were cut into 5-µm-thick sections. The sections were incubated overnight (18 h) at 4 °C with the following primary monoclonal antibodies: anti-CD8 (BS-0648R; Bioss < Bioss Antibodies, Woburn, MA, USA), anti-IL-17 (ab79056; Abcam), and anti-pSTAT3 (Y705, ab76315; Abcam). After washing, sections were treated with a secondary antibody using the Dako Envision + System-HRP Labeled Polymer Anti-Rabbit (K400311-2; Agilent Technologies, Santa Clara, CA, USA) for 30 min at room temperature. Positive signals were visualized with a DAB substrate kit (K346811-2; Agilent Technologies). For analysis, two representative images (400× magnification) were captured from each stained slide, and the number of positive cells at the dermal–epidermal junction was quantified using ImageJ (NIH, Bethesda, MD, USA).

### In vitro evaluation

Splenocytes were isolated from SKG mice by gently mincing freshly harvested spleens on a sterile teasing slide to obtain single-cell suspensions. Erythrocytes were lysed with ACK lysis buffer, and cell suspensions were filtered through a 40 μm cell strainer to remove debris. After washing, immune cell concentration was adjusted to 1 × 10^6^ cells/ml per well for flow cytometry assays, or to 1 × 10^7^ cells/ml per well for western blot analysis. Cells were then cultured and stimulated for 72 h with anti-CD3 (2 µg/ml, 553057; BD Biosciences, USA), anti-CD28 (2 µg/ml, 553294; BD Biosciences), and recombinant IL-6 (10 ng/ml, 406-ML-025; R&D Systems) to drive psoriatic differentiation via STAT3 phosphorylation. For western blot analysis, cells were restimulated under the same conditions for an additional 1 h prior to harvesting. To assess the impact of NGO on pSTAT3 signaling, cells were treated with NGO (31NM-0008; INBCT Co., Ltd.) and NXB (Biotin labeled NGO) (31CT-0001; INBCT Co., Ltd.) at concentrations of 1 and 10 µg/ml during stimulation. Following incubation, both cells and culture supernatants were harvested for downstream analyses of gene transcription and protein expression.

### Western blotting

Single-cell suspensions were generated from the spleens of acute psoriasis model mice. Cells were lysed in 50 µL of RIPA Lysis and Extraction Buffer (89901; Thermo Fisher Scientific, Rockford, IL, USA) to prepare whole protein lysates. The protein concentration was quantified by bicinchoninic acid (BCA) assay (23235; Thermo Fisher Scientific). Equal amounts of total protein were separated by 10–12% SDS-PAGE and transferred onto nitrocellulose membranes (Amersham Pharmacia, Uppsala, Sweden). After blocking, the membranes were incubated at room temperature for 35 min with primary antibodies against pSTAT3 (Ser727) (#9134; Cell Signaling Technology, Danvers, MA, USA), pSTAT3 (Tyr705) (#9131; Cell Signaling Technology), STAT3 (#9139; Cell Signaling Technology), and GAPDH (ab181602; Abcam), all diluted in 0.4% skim milk in 1× Tris-buffered saline with 0.1% Tween-20 (TBS-T). The membranes were washed and subsequently incubated with HRP-conjugated secondary antibodies for 20 min at room temperature. Target bands were visualized by enhanced chemiluminescence. Quantitative analysis of band intensity was conducted using ImageJ (NIH), and the results were expressed as the ratio of target protein to GAPDH.

### Mitochondrial respiration and cellular energy profiling

Cellular metabolic profiling was performed by measuring the mitochondrial oxygen consumption rate (OCR) in splenic immune cells isolated from SKG mice and human-derived PBMCs, adjusted to 1 × 10^6^ cells per well, and stimulated 72 h with anti-CD3, anti-CD28, and recombinant IL-6, or recombinant IL-23 (10 ng/ml, 1290-IL-010, R&D Systems) using an XF24 Extracellular Flux Analyzer (Seahorse Bioscience, Chicopee, MA, USA). To assess mitochondrial respiration, sequential injections were administered as follows: 4 µM oligomycin to determine ATP-coupled respiration; 3 µM carbonyl cyanide-4-(trifluoromethoxy) phenylhydrazone (FCCP) to measure maximal respiratory capacity; and a combination of 2 µM rotenone and 2 µM antimycin A to inhibit complexes I and III for assessment of non-mitochondrial respiration. Basal respiration was calculated as the difference between baseline OCR and rotenone/antimycin A OCR, ATP-linked respiration was determined following addition of oligomycin, and maximal respiration was measured as the OCR after FCCP injection minus non-mitochondrial OCR. All measurements and subsequent analyses were performed using Wave software (Agilent Technologies).

### Mitochondrial analysis by flow cytometry

Splenic immune cells and patient-derived PBMCs were seeded at 5 × 10^5^ cells per well and stimulated 72 h with anti-CD3, anti-CD28, and recombinant IL-6. The mitochondrial ROS level was measured by MitoSOX (M36008; Thermo Fisher Scientific) staining, and analyzed by flow cytometry (FACSCalibur; BD Biosciences).

### Flow cytometry

Single-cell suspensions were prepared from spleens isolated from mice with acute, psoriasis-like inflammation, and plated at a density of 1 × 10^6^ cells per well. Cells were stimulated for 4 h with phorbol 12-myristate 13-acetate (PMA, P8139; Sigma-Aldrich, Sigma-Aldrich, St. Louis, MO, USA) and ionomycin (I0634; Sigma-Aldrich). For analysis of effector T cell populations, cells were stained with the following antibodies: CD4-PC5.5 (#45-0042-82; eBioscience, San Diego, CA, USA), IL-17-FITC (506910; BioLegend, San Diego, CA, USA), and IFN-γ-APC (505810; BioLegend) for effector CD4^+^ T cells; CD8-PB 450 (560409; BD Biosciences), IL-17-FITC (506910; BioLegend), and IFN-γ-APC (505810; BioLegend) for effector CD8^+^ T cells. To identify TRM cells, additional staining was performed with CD103-APC-A700 (56–1031-82; eBioscience) and CD69-PE (12–0691-82; eBioscience) in combination with the abovementioned T cell antibody panels. Regulatory T cells (Tregs) were identified using antibodies against CD4-PC5.5 (#45-0042-82; eBioscience), CD25-APC (#102012; BioLegend), and FOXP3-PE (#12–5773-82; eBioscience). Data acquisition and analysis were conducted using a FORTESSA flow cytometer (BD Biosciences). Representative full gating strategy used for the identification and analysis of tissue-resident memory T (TRM) cells and their cytokines are provided in the Supplementary Materials.

### Patient-derived specimen analysis

Peripheral blood mononuclear cells (PBMCs) were obtained from anticoagulated whole blood samples from patients with psoriasis (*n* = 6; PASI > 15 and body surface area involvement > 10%) treated at St. Mary’s Hospital, Seoul, South Korea (IRB No. KC17TNSI0237, Approval date: 2024.04.05). PBMCs were isolated by density gradient centrifugation using Ficoll-Paque PLUS (#17–1440-03; Cytiva, Marlborough, MA, USA). Freshly isolated cells were immediately subjected to immunophenotyping to delineate immune cell populations associated with disease activity. All procedures involving human subjects were conducted in accordance with the ethical principles of the institutional and/or national research committees and the 1964 Declaration of Helsinki, as well as its subsequent revisions.

### Statistical analysis

All statistical analyses were carried out using GraphPad Prism 8. Data are presented as mean ± SD. Two-tailed p-values less than 0.05 were considered statistically significant. For comparisons among more than two groups, two-way ANOVA was performed followed by Bonferroni post hoc correction for multiple comparisons. For nonparametric multiple-group comparisons, the Kruskal–Wallis test with Dunn’s post hoc test was applied. Two-group comparisons were evaluated using the Mann–Whitney U test. Statistical significance is denoted as *P < 0.05, **P < 0.01, ***P < 0.001, and ****P < 0.0001.

## Results

### Physicochemical characterization and biocompatibility of NGO

**Fig. 1:**
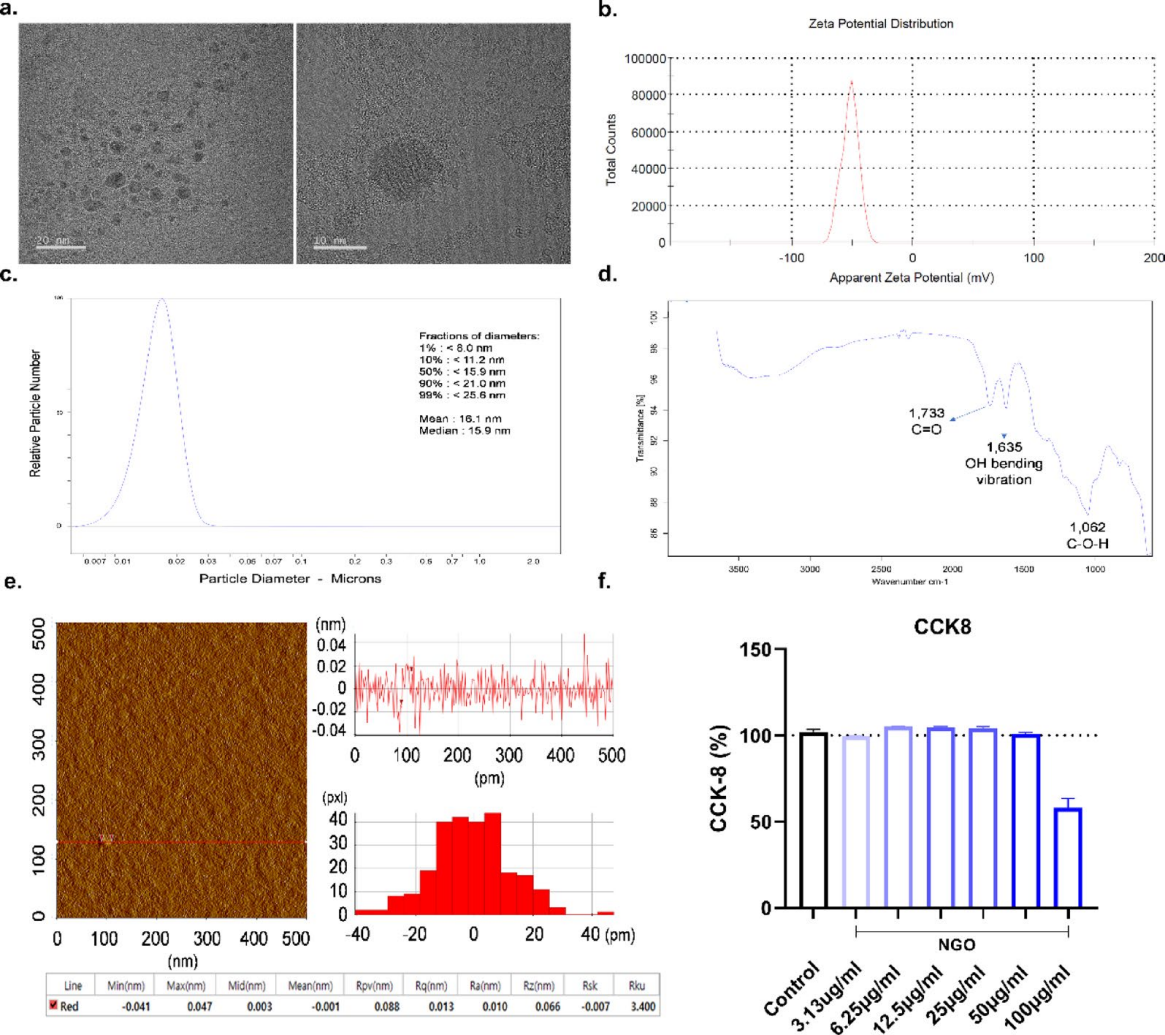
Physicochemical characterization of nano-sized graphene oxide (NGO) **(A)** Representative transmission electron microscopy images showing the ultrathin nature (left) and lateral (right) size. **(B)** A single peak was observed at − 51.7 mV, with a mean value of − 53.1 mV. **(C)** Particle size distribution indicating a narrow particle size distribution with most NGO nanosheets in the range of ~ 20–40 nm. **(D)** FTIR spectrum of NGO demonstrating characteristic oxygen-containing functional groups, including C = O (1733 cm^− 1^), O–H bending (1635 cm^− 1^), and C–O (1062 cm^− 1^). **(E)** Atomic force microscopy topography of GO (left) with line profile, surface roughness, and thickness distribution analysis (right), confirming nanosheet thickness of ~ 1–2 nm. **(F)** Treatment of HaCaT cells with NGO (3.13–50 µg/ml, 24 h) did not induce cytotoxicity as determined by CCK-8 assay

We performed a series of structural and spectroscopic analyses to verify the physicochemical properties of NGO. TEM confirmed the ultrathin nature and sheet-like morphology of NGO flakes (Fig. 1A). The surface charge of the sample was analyzed using dynamic light scattering–based zeta potential measurements. The distribution profile exhibited a single, sharp peak centered at − 51.7 mV, and no additional populations were detected (Fig. 1B). The mean (SD) zeta potential was calculated to be − 53.1 (3.94) mV, confirming the homogeneity and strong negative surface charge of the dispersion. Disc stack centrifugation demonstrated that most NGO particles were around ~ 20–40 nm in diameter, a relatively narrow size distribution (Fig. 1C). Fourier-transform infrared (FTIR) spectroscopy further validated the oxygen-containing functional groups on the NGO surface, revealing distinct peaks corresponding to C = O stretching (1733 cm^− 1^), O–H bending (1635 cm^− 1^), and C–O stretching (1062 cm^− 1^) (Fig. 1D). AFM imaging revealed the topographic features of NGO, displaying uniform sheet thickness at the nanoscale level (Fig. 1E, left). Line profile and height distribution analyses confirmed an average thickness of approximately 1–2 nm, consistent with single- to few-layered NGO nanosheets (Fig. 1E, right). In addition, the cytotoxic effects of NGO were evaluated in HaCaT using the WST-8 assay after 24 h of treatment at concentrations of 3.13–50 µg/ml (Fig. 1F). None of the compounds tested induced cytotoxicity across the concentration range, with cell viability consistently maintained at or above control levels. Taken together, these analyses confirmed the successful synthesis and structural integrity of NGO with abundant oxygen functional groups, uniform nanosheet morphology, and stable dispersion properties suitable for subsequent biological studies.

### NGO-mediated modulation of mitochondrial homeostasis and STAT3–IL-17 signaling in immune cells

**Fig. 2:**
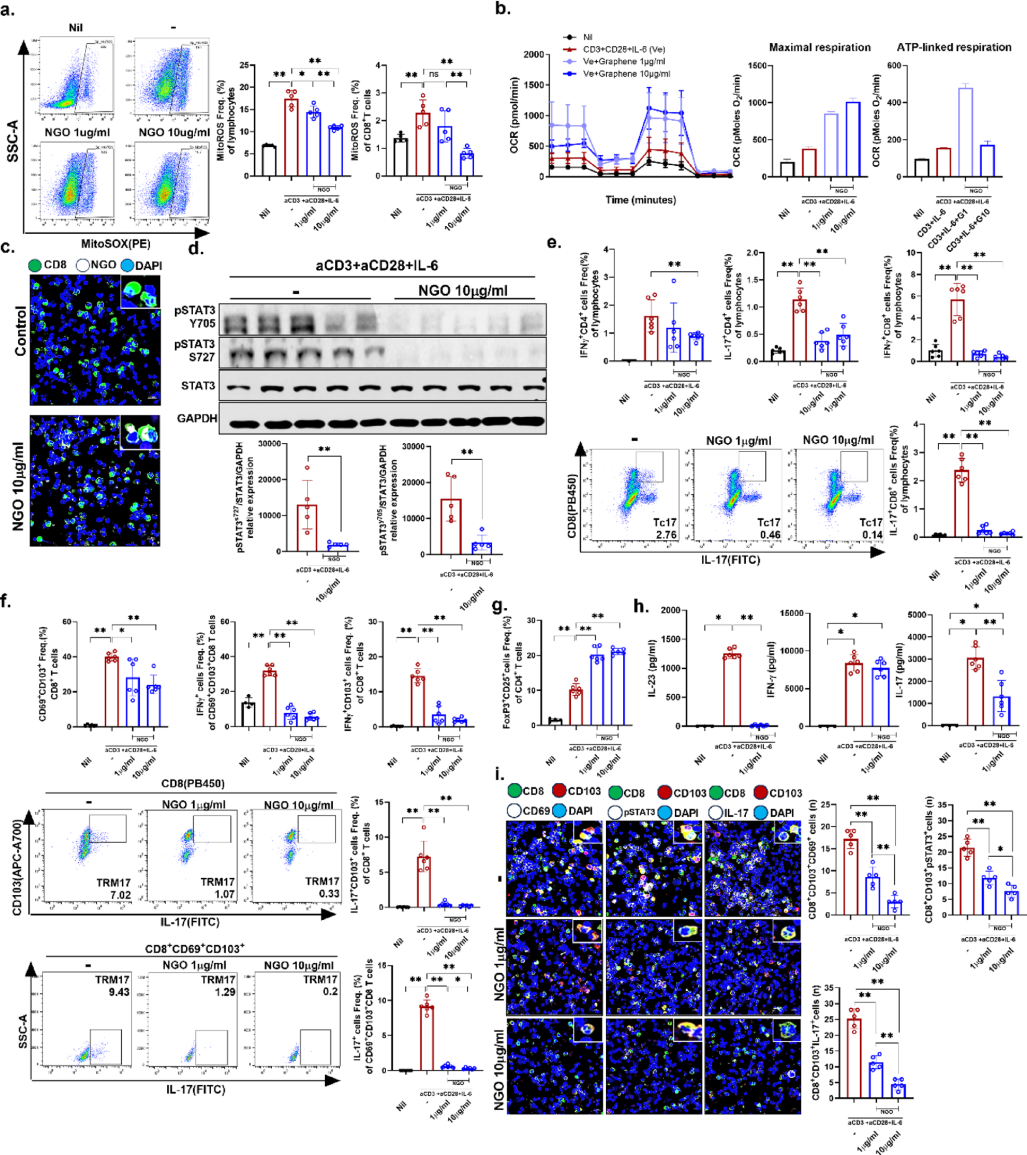
Nano-sized graphene oxide impaired mitochondrial function and potently suppressed pSTAT3 signaling to regulate pathogenic tissue-resident memory cell differentiation and function in vitro **(A)** Mitochondrial ROS production in splenic immune cells and CD8^+^ T cells (stimulated with anti CD3 2 µg/ml, aCD28 2 µg/ml, IL-6 10ng/ml) was assessed after nano-sized graphene oxide (NGO) treatment using MitoSOX staining. **(B)** The mitochondrial oxygen consumption rate in splenic immune cells, under conditions mentioned above, was measured over time following NGO exposure. **(C)** Immunofluorescence imaging (200x) was performed to examine the distribution of splenic CD8^+^ T cells in the presence or absence of biotinylated NGO 10 µg/ml (CD8: green; NGO: white; DAPI: blue). **(D)** Western blotting analysis was used to evaluate phosphorylation of STAT3 at Ser727 and Tyr705, and STAT3 protein expression in NGO-treated splenic immune cells, stimulated under conditions mentioned above. **(E**–**G)** Flow cytometry was performed in splenic immune cells at same stimulation conditions to determine the frequencies of IFN-γ^+^ and IL-17^+^ CD8^+^CD103^+^ TRM-like T cells, as well as CD69^+^CD103^+^ TRM cell subsets, in the presence or absence of NGO and regulatory T (Treg) cells. **(H)** Cytokine concentrations associated with psoriasis in splenic immune cell culture supernatants were measured by ELISA following treatment with 1 µg/ml NGO. **(I)** Multiplex immunofluorescence staining in splenic immune cells at same stimulation conditions was used to detect CD8^+^CD103^+^, CD69^+^, pSTAT3^+^, and IL-17^+^ cells, followed by quantitative analysis (CD8: green; CD103: red; CD69, pSTAT3, IL-17: white; DAPI: blue). *n* = number. All values are shown as the mean ± SD (*n* = 6 for aCD3 + aCD28 + IL-6 condition, *n* = 6 for NGO 1 µg/ml, *n* = 6 for NGO 10 µg/ml). Each experiment was repeated three times. Statistical significance was determined by non-parametric Mann–Whitney U test for pairwise comparisons and one-way ANOVA using Kruskal-Wallis test for multiple group comparisons. *P < 0.05, **P < 0.01, ***P < 0.001, ****P < 0.0001

To evaluate the effects of NGO on mitochondrial homeostasis within immune cells, we performed in vitro analyses of mitochondrial function and phenotype using splenic immune cell preparations with psoriatic pSTAT3–IL-17^+^ T cell differentiation conditions. NGO treatment modulated mitochondrial reactive oxygen species (mtROS) levels not only in total splenic lymphocytes but also specifically in CD8^+^ T cell subsets (Fig. 2A). Restoration of mitochondrial function was evidenced by normalization of aberrantly elevated OCR at both NGO concentrations tested, as determined by extracellular flux analysis (XF24 Extracellular Flux Analyzer; Seahorse Bioscience), which indicated reduced metabolic activity of IL-17^+^ cells (Fig. 2B). To effectively demonstrate cellular internalization, the intracellular uptake of NGO was visually assessed using a relatively high dose of 10 µg/ml. Upon activation of T cells with subsequent pSTAT3–IL-17 pathway induction, administration of biotin-linked NGO (10 µg/ml) resulted in efficient engraftment of NGO within CD8^+^ T cells, as confirmed by detection of streptavidin-positive cells, demonstrating successful intracellular delivery (Fig. 2C). Under identical stimuli, the levels of both forms of pSTAT3 (Ser727 and Tyr705) were markedly decreased by treatment with 10 µg/ml NGO (Fig. 2D), with this concentration selected due to limited splenocyte availability to ensure sufficient cell numbers for reliable western blot analysis. Moreover, NGO demonstrated a dose-dependent inhibitory effect on both IFN-γ– and IL-17–producing CD4^+^ and CD8^+^ T cells. (Fig. 2E). Notably, the frequencies of the TRM cell subsets (CD69^+^CD103^+^CD8^+^) and IFN-γ/IL-17–producing CD103^+^CD8^+^ and CD69^+^CD103^+^CD8 TRM cells were significantly reduced following NGO exposure in a dose-dependent manner (Fig. 2F). In contrast, the percentage of Tregs increased with increasing NGO concentration (Fig. 2G). The full gating strategy is provided in the supplementary materials. Analysis of cytokine secretion further demonstrated that NGO suppressed the production of psoriasis-related proinflammatory cytokines, including IL-23 and IL-17, at 1 µg/ml, while the reduction in IFN-γ levels was not substantial. This concentration that was selected based on corresponding efficient effects already observed in both FACS and confocal microscopy analyses. (Fig. 2H). Taken together, these observations show that NGO treatment restored mitochondrial homeostasis by reducing excessive mtROS production and normalizing aberrantly elevated OCR in splenic immune cells. This mitochondrial regulation was closely associated with suppression of pSTAT3–IL-17 signaling, leading to a marked reduction of pathogenic CD8^+^ TRM cell subsets and highlighting its potential as a novel immunoregulatory strategy (Fig. 2I).

### Therapeutic effects of NGO in a psoriasis mouse model via suppression of STAT3–IL-17 CD8^+^ TRM cells

**Fig. 3:**
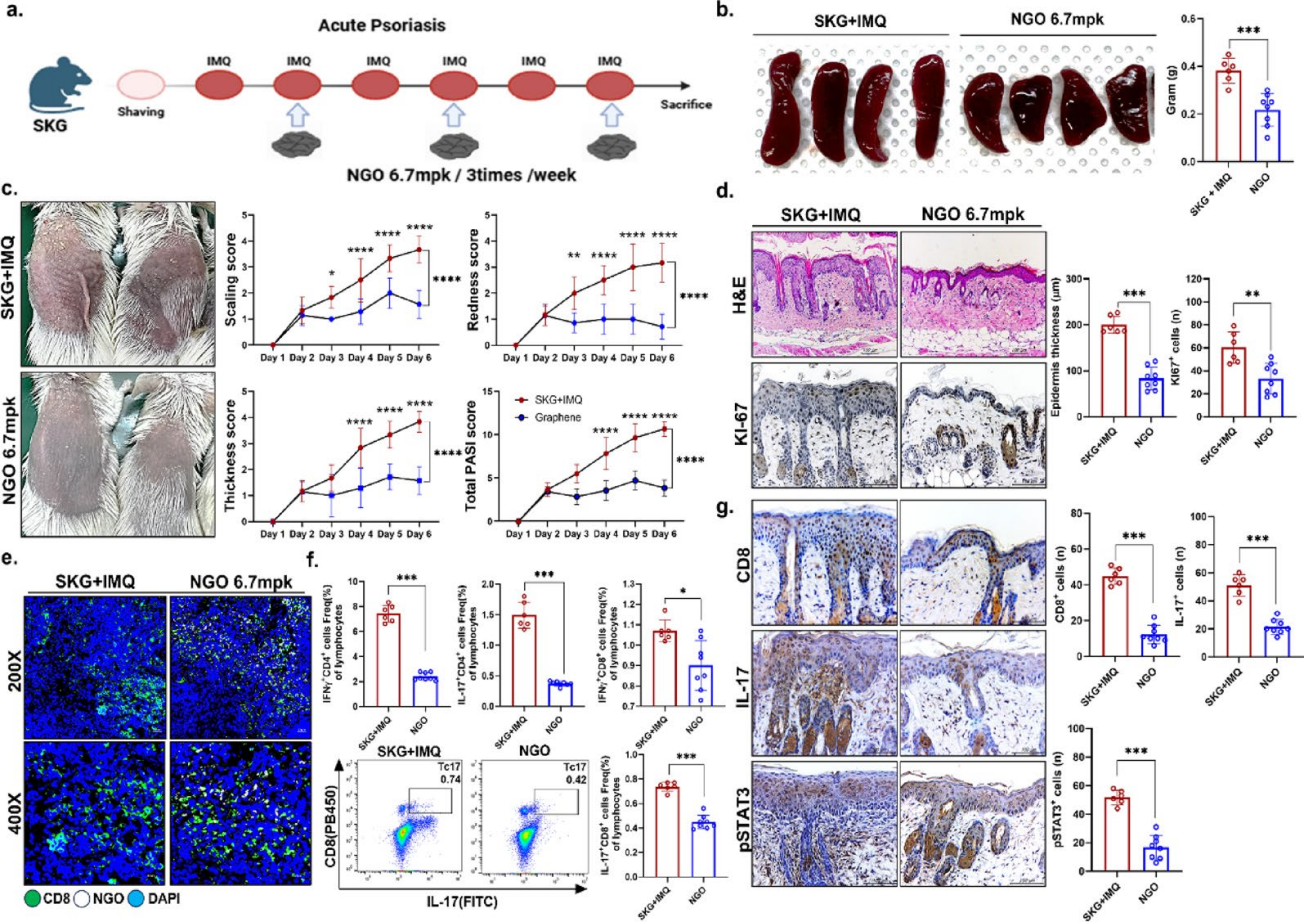
Nano-sized graphene oxide (NGO) attenuated psoriatic skin inflammation by suppressing pSTAT3–IL-17 immune responses in vivo **(A)** Schematic overview of the experimental protocol. Psoriasis-like skin inflammation was induced in mice using imiquimod, followed by repeated NGO administration. **(B)** Measurement of spleen size and weight in control and NGO-treated mice. **(C)** Clinical scoring of skin scaling, erythema, thickness, and the Psoriasis Area and Severity Index (PASI) in control and NGO-treated mice. **(D**,** G)** Histological and immunohistochemical staining to determine epidermal thickness and Ki-67^+^, CD8^+^, IL-17^+^, and pSTAT3^+^ cells in skin lesions. **(E)** Representative immunofluorescence images of spleen sections from each group show CD8^+^ T cells, biotin-labeled NGO, and nuclei in the spleen at magnifications of 200× and 400× (CD8: green; NGO: white; DAPI: blue). **(F)** Flow cytometric analysis of IFN-γ^+^ and IL-17^+^ CD4^+^ and CD8^+^ T cells in the spleen. *n* = number. All data are shown as the mean ± SD (*n* = 6 for SKG + IMQ, *n* = 8 for with NGO). Each experiment was repeated three times. Statistical significance was determined by two-way ANOVA with Bonferroni test in multiple groups and non-parametric Mann–Whitney U test for pairwise comparisons. *P < 0.05, **P < 0.01, ***P < 0.001, ****P < 0.0001

**Fig. 4:**
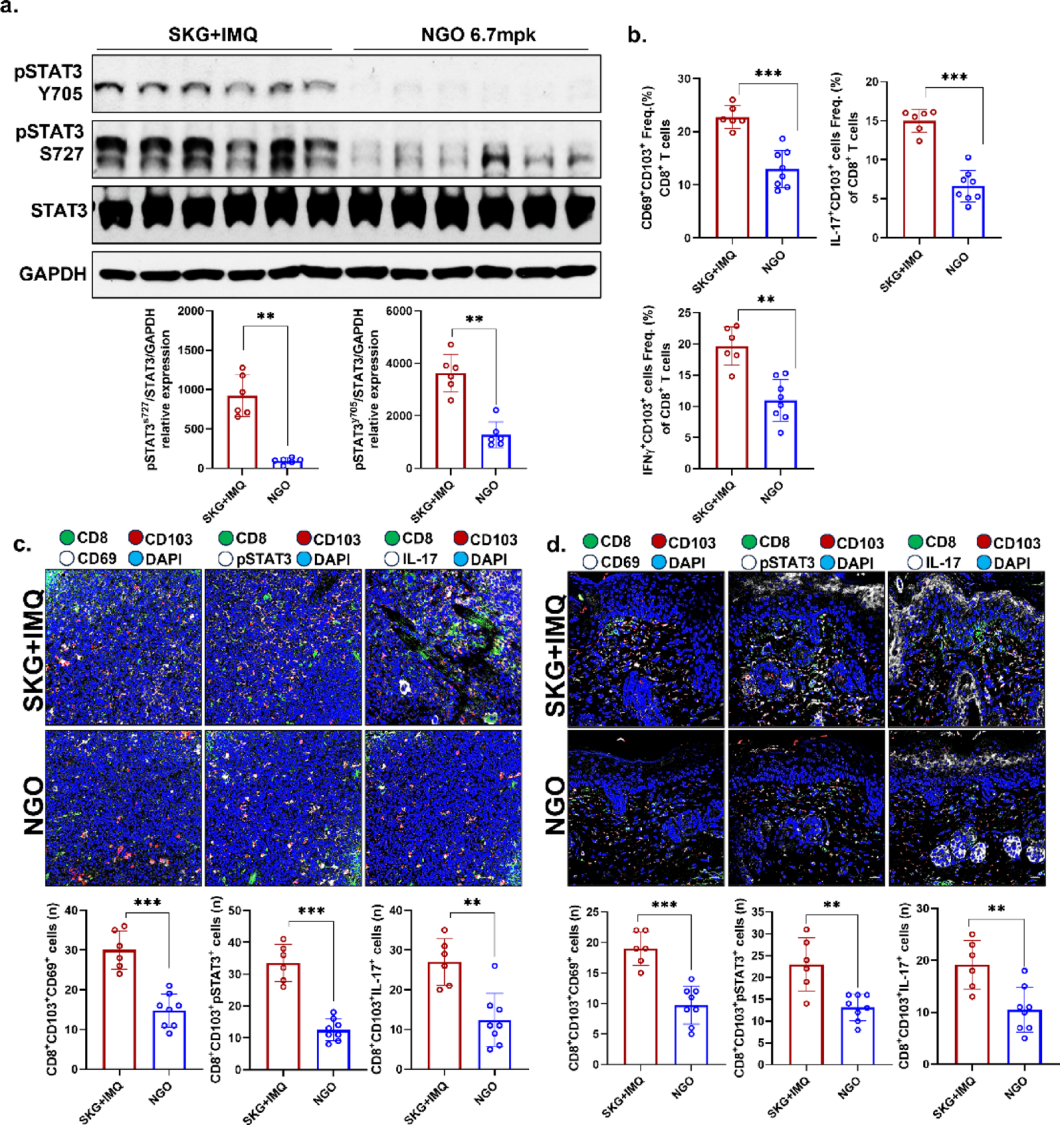
Nano-sized graphene oxide (NGO) attenuated psoriatic inflammation by inhibiting STAT3 activation and suppressing proinflammatory CD8^+^ tissue-resident memory (TRM) T cell accumulation in tissue **(A)** Western blotting analysis of splenic immune cells from psoriatic mice revealed that NGO treatment markedly inhibited phosphorylation of STAT3 at both Ser727 and Tyr705, quantified relative to total STAT3 and GAPDH (*n* = 6 for SKG + IMQ, *n* = 6 for with NGO in western blot). **(B)** Flow cytometry showed that the frequency of activated CD69^+^CD103^+^CD8^+^ T cells, as well as IFN-γ^+^ and IL-17^+^ CD103^+^CD8^+^ T cell subsets, indicating suppression of key proinflammatory TRM cell populations in splenic immune cells from NGO treated psoriatic mice. **(C)** Multiplex immunofluorescence imaging and quantification demonstrated significant decreases in CD8^+^CD103^+^CD69^+^ TRM cells, CD8^+^CD103^+^pSTAT3^+^, and CD8^+^CD103^+^IL-17^+^ cells in spleen tissues after NGO treatment (CD8: green; CD103: red; CD69, pSTAT3, IL-17: white; DAPI: blue). **(D)** Similar immunofluorescence analyses of skin lesions supported reduced presence of TRM cell markers and effector cytokines with NGO treatment, confirmed by quantitative positive cells counting (CD8: green; CD103: red; CD69, pSTAT3, IL-17: white; DAPI: blue). *n* = number. All data are shown as the mean ± SD (*n* = 6 for SKG + IMQ, *n* = 8 for with NGO in flow cytometry and tissue data). Each experiment was repeated three times. Statistical significance was determined by non-parametric Mann–Whitney U test for pairwise comparisons. *P < 0.05, **P < 0.01, ***P < 0.001

An acute psoriasis model was induced in SKG mice by treatment with IMQ. NGO was administered simultaneously at a dose of 6.7 mg/kg intraperitoneally three times per week, followed by sacrifice at the end point (Fig. 3A). Compared to the disease control group, the spleen of NGO-treated mice exhibited a marked reduction in size (Fig. 3B). Therapeutic effects of NGO were also observed in clinical assessments, as evidenced by significant improvements in erythema, scaling, and skin-thickness scores (Fig. 3C). Histopathological analysis further revealed a reduction in epidermal thickness and attenuation of keratinocyte proliferation in the skin lesions of NGO-treated mice (Fig. 3D). To verify further NGO infiltration in vivo, biotin-labeled NGO was injected intraperitoneally into animal models. An increase in the streptavidin-positive rate was observed, indicating that NGO was effectively internalized into CD8^+^ T cells within the spleen (Fig. 3E). Immunophenotypic analysis of splenic immune cells demonstrated a decrease in the frequency of IFN-γ/IL-17–producing CD4^+^ and CD8^+^ T cell subsets following NGO treatment (Fig. 3F). Finally, analysis of skin lesions showed that the abundance of CD8^+^ T cells and the expression of pSTAT3 and IL-17 were also significantly reduced in the NGO treatment group, consistent with the findings in splenic immune cells (Fig. 3G). Evaluation of splenic immune cells after sacrifice showed marked suppression of both forms of pSTAT3 (Ser727 and Tyr705) in the NGO treatment group (Fig. 4A). Furthermore, NGO administration effectively inhibited the generation of splenic CD8^+^ TRM cells, as well as the IFN-γ/IL-17–producing CD8^+^ TRM cell subsets (Fig. 4B). Consistent with these observations, immunohistochemical analysis of splenic tissue demonstrated reduced levels of CD8^+^ TRM cells and a decrease in pSTAT3–IL-17–expressing CD8^+^ TRM cells. A similar trend was observed in skin lesions from the psoriasis model, supporting the potential therapeutic effect of NGO in attenuating pathogenic T cell responses (Fig. 4C, D).

### NGO restored mitochondrial function and suppressed the STAT3–IL-17 axis in immune cells from psoriasis patients

**Fig. 5:**
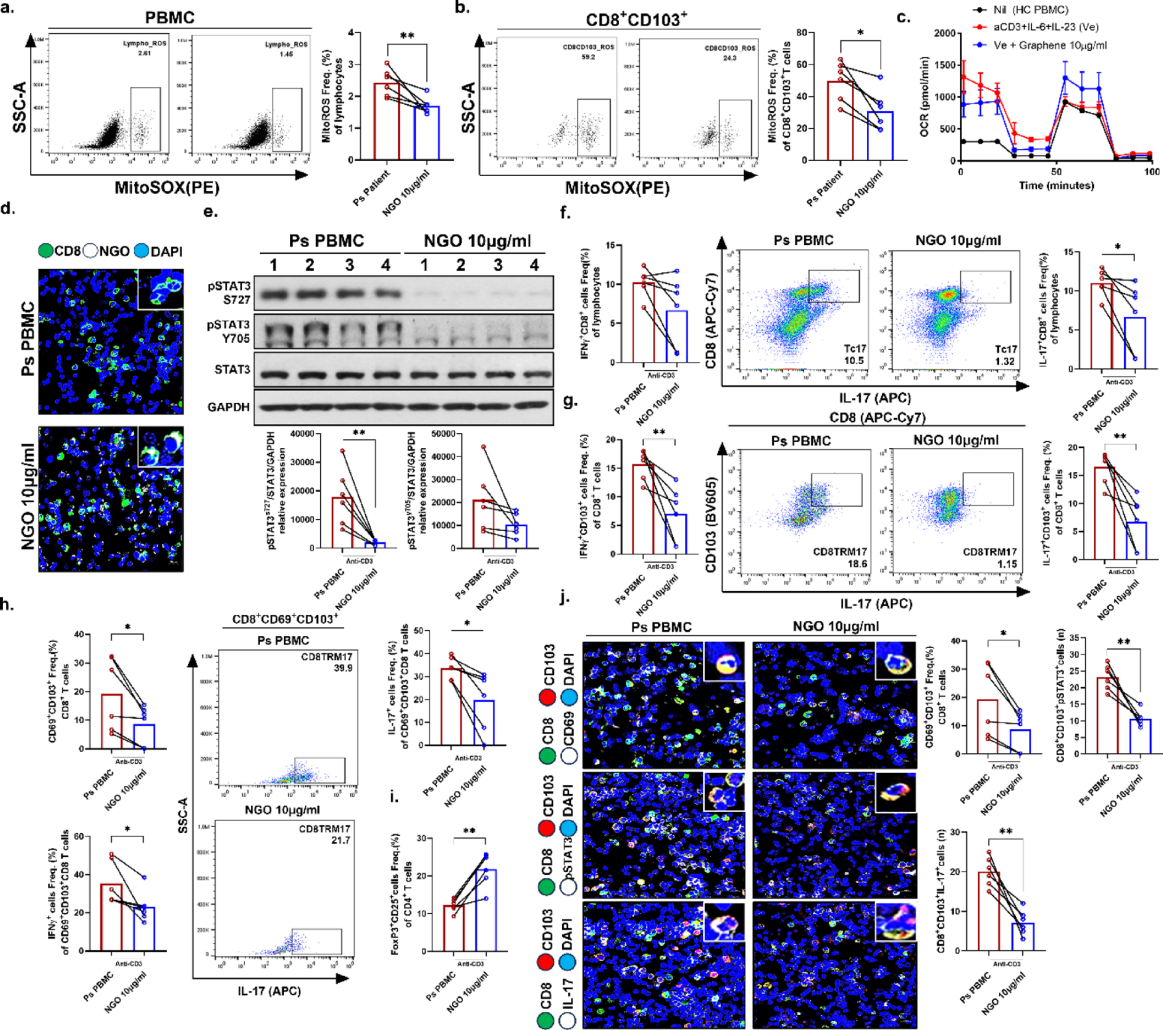
Nano-sized graphene oxide (NGO) restored mitochondrial function and suppressed proinflammatory tissue-resident memory CD8^+^ T cells in the peripheral blood of patients with psoriasis by regulating the pSTAT3–IL-17 axis.**(A, B) **Mitochondrial ROS (MitoSOX staining) was measured in whole peripheral blood mononuclear cells (PBMCs) and CD8^+^CD103^+^ T cells from patients with psoriasis following NGO treatment, highlighting altered mitochondrial function.** (C) **The mitochondrial oxygen consumption rate was analyzed in healthy control PBMCs under conditions for psoriatic IL-17–expressing T cell differentiation (stimulated with anti CD3 2μg/ml, aCD28 2μg/ml, IL-23 10ng/ml).** (D) **Immunofluorescence imaging showed the co-localization and frequency of CD8^+^ T cells in the presence or absence of NGO 10μg/ml (CD8: green; NGO: white; DAPI: blue), with marked reduction upon NGO treatment in psoriasis patients’ PBMCs.** (E) **Western blotting and quantification demonstrated significant inhibition of STAT3 phosphorylation (Ser727 and Tyr705) in psoriasis patients’ PBMCs treated with NGO 10μg/ml.** (F, G) **Flow cytometric analysis showed that the population of IFN-γ^+^ or IL-17^+^ CD4^+^/CD8^+^ T cells and CD8^+^CD103^+^ tissue-resident memory (TRM)-like T cells, as well as their cytokine production in psoriasis patients’ PBMCs.** (H) **The frequency of CD8^+^CD69^+^CD103^+^ and IFN-γ^+^ or IL-17^+^ TRM cell subsets, as determined by flow cytometry in psoriasis patients’ PBMCs.** (I) **Regulatory T cell (Treg, Foxp3^+^CD25^+^CD4^+^) frequency assessed by flow cytometry in psoriasis patients’ PBMCs.** (J) **Multiplex immunofluorescence (200ⅹ) of patient-derived immune cells further confirmed decreased TRM cell marker CD8^+^CD103^+^, CD69^+^, pSTAT3^+^, and IL-17^+^ expression after NGO treatment, and quantitative analysis supported significant reductions in psoriasis patients’ PBMCs (CD8: green; CD103: red; CD69, pSTAT3, IL-17: white; DAPI: blue). Ps: psoriatic, HC: healthy control. *n* = number. All data are shown as the mean ± SD (n=6 for Psoriasis PBMC, n=6 for with NGO 10 μg/ml). Detailed patient information is described in the supplementary materials. Each experiment was repeated two times. Statistical significance was determined by non-parametric Mann–Whitney U test for pairwise comparisons. * *P* < 0.05,**P < 0.01, ***P < 0.001,****P < 0.0001.

Finally, the therapeutic potential of NGO was evaluated in vitro using PBMCs from patients with psoriasis, focusing on mitochondrial function. Due to limited availability of patient-derived immune cells, experiments were conducted using a single, relatively high dose of 10 µg/ml to ensure sufficient cell numbers and effective results. Treatment with NGO at 10 µg/ml significantly reduced mtROS levels in PBMCs from the blood of psoriasis patients as well as in CD103^+^CD8^+^ T cells (Fig. 5A, B). Furthermore, an increase in OCR level was observed upon NGO treatment in healthy control PBMCs under IL-17–expressing T cell differentiation conditions, indicating that the IL-17–associated mitochondrial metabolic phenotype was effectively suppressed (Fig. 5C). Consistent with the findings in murine splenic immune cells, biotin-labeled NGO was efficiently internalized by patient-derived CD8^+^ T cells (Fig. 5D). NGO treatment also led to effective suppression of both forms of pSTAT3 (Ser727 and Tyr705) in immune cells from patients with psoriasis (Fig. 5E). Furthermore, NGO treatment markedly suppressed IL‑17 production and, to a lesser extent, reduced IFN‑γ expression in CD8^+^ T cells but not in CD4^+^T cells and exerted a similar suppressive effect on the CD103^+^CD8^+^ TRM cell subset (Fig. 5F, G). The full gating strategy is provided in the supplementary materials. Notably, there were marked reductions in the frequencies of CD69^+^CD103^+^CD8^+^ TRM cells as well as IFN-γ/IL-17–producing CD69^+^CD103^+^CD8^+^ TRM cells following NGO exposure (Fig. 5H). In contrast, Tregs showed an increasing trend following NGO treatment (Fig. 5I). Visualization of the reduction in CD8^+^ TRM cell numbers further confirmed the marked inhibition of CD69^+^CD103^+^CD8^+^ TRM cells and the suppression of pSTAT3–IL-17–expressing CD8 TRM cells by NGO in patient-derived immune cells (Fig. 5J).

Taken together, these observations show that NGO restored mitochondrial function, inhibited STAT3–IL-17 signaling, and reduced pathogenic Th17, Tc17 and CD8^+^ TRM cell numbers, thereby suppressing psoriatic inflammation and promoting Treg differentiation. These findings highlight the therapeutic potential of NGO for psoriasis, as confirmed in both the models and the patient-derived cells.

## Discussion

In this study, NGO was shown to be a biocompatible nanomaterial with favorable properties for biomedical applications. Structural analyses, including TEM, AFM, and CPS, confirmed that NGO has a thin, sheet-like morphology with lateral sizes in the nanometer range and an average thickness of 1–2 nm, consistent with single- to few-layer nanosheets. FTIR further demonstrated abundant oxygen-containing functional groups, confirming the chemical integrity of the material. Importantly, zeta potential analysis revealed a highly negative surface charge (~ − 53 mV), indicative of excellent colloidal stability, which supports dispersion in aqueous environments and prevents aggregation, both properties essential for reproducible biological activity.

These physicochemical properties provide the foundation for the bioactivity of NGO observed in our immunological studies. The nanoscale dimensions and sheet-like morphology facilitate cellular uptake and mitochondrial interactions [23], while oxygenated functional groups enable redox modulation and compatibility with biological systems [24]. High colloidal stability further ensures reproducibility of biological effects by preventing aggregation. Therefore, the characterization of NGO not only validated the quality of the material but also established a mechanistic rationale for its therapeutic potential in the treatment of immune-mediated diseases.

We identified NGO as an immunomodulatory agent capable of attenuating psoriatic inflammation by suppressing a broad array of pathogenic cell subsets, including IL17 expressing CD4^+^ and CD8^+^ T cells, IL23 producing cells, and TRM cells that collectively serve as central drivers of psoriasis pathogenesis. NGO consistently reduced mtROS levels, normalized oxidative respiration, decreased phosphorylation of STAT3 at Ser727 and Tyr705, which are key sites regulating inflammatory transcriptional activity, and suppressed IL17 production in multiple complementary systems, including in vitro T cell polarization, the IMQ induced psoriasis model, and patient derived peripheral immune cells. These molecular and cellular changes were accompanied by reductions in CD8^+^CD69^+^CD103^+^ TRM/TRM17 subsets and increases in Treg populations, translating into histological and clinical improvement in vivo.

Phosphorylation of STAT3 Tyr705 and Ser727 plays a pivotal role in promoting IL-17 expression, functioning as a key transcriptional activator that drives Th17 cell differentiation and proinflammatory cytokine production [25–27]. In contrast, a portion of STAT3 Ser727 localizes to mitochondria, where it modulates the activity of electron transport chain complexes I and II, thereby supporting oxidative phosphorylation and maintaining cellular respiration [28, 29]. Emerging evidence indicates that mitochondrial-localized phosphorylated Ser727 may attenuate Th17 cell differentiation and IL-17 production [30, 31]. Conversely, under conditions of heightened IL-17 signaling and STAT3 nuclear activation, there is associated mitochondrial dysfunction manifested as elevated mitochondrial ROS, reduced OCR, and impaired metabolic performance [32]. Disturbances in mitochondrial activity also directly impair STAT3 signaling, and ETC-derived ROS further reinforce this axis. For example, H_2_O_2_ rapidly activates STATs, while oxidation of protein tyrosine phosphatases prolongs STAT3 phosphorylation [13, 33, 34]. These links provide a mechanistic rationale for simultaneously attenuation of nuclear STAT3 Ser727, Tyr705 activation by lowering mtROS, thereby limiting IL-17 production rather than mitochondria specific localized STAT3. Consistent with this mechanism, our data demonstrate that NGO reduced mtROS and normalized respiration, leading to decreased nuclear STAT3 phosphorylation and IL-17 output.

Pathogenic Th17 differentiation is tightly coupled to oxidative phosphorylation (OXPHOS), and interference with mitochondrial respiration disrupts STAT3-dependent cell fate decisions [30, 35]. In addition to Th17, the emergence and persistence of skin TRM cells are reinforced by cytokines that signal through STAT3—most notably IL-23, which activates STAT3 via JAK2/TYK2 [3, 36]. Genetic and infection models have further demonstrated that STAT3 signaling is essential for memory T cell formation and maturation, implying that TRM cells also rely on STAT3-dependent programs [37]. Consistent with these results, our findings show that NGO normalized respiration and reduced STAT3 phosphorylation while selectively depleting IL-17–dominant TRM cell populations. While IFN-γ production is primarily regulated by STAT4, distinct from STAT3-dependent IL-17 expression as shown in previous studies our data align with this mechanism showing a more pronounced effect of NGO on IL-17 suppression compared to its effect on IFN-γ levels [38]. Skin-resident CD8^+^ TRM exhibit functional heterogeneity, with IL-17-producing subsets being significantly enriched in psoriatic lesions. These IL-17-producing TRM cells, rather than IFN-γ producers, represent a more critical pathogenic target in psoriasis, consistent with the stronger suppression of IL-17 observed in our study, which underscores the specificity of NGO’s action on the STAT3–IL-17 axis central to psoriatic inflammation [39].

NGO-based materials have also been shown to suppress STAT3-driven pathogenesis in a range of other disease models. In retinal angiogenesis, NGO quantum dots decreased pathological neovascularization by blocking a pSTAT3/periostin/ERK cascade in an oxygen-induced retinopathy model, providing in vivo evidence that NGO can attenuate STAT3 signaling [40]. In oncology, functional NGO oxide has been used to deliver STAT3-targeting siRNA, thereby reducing tumor growth [41]. In breast cancer, in which IL-6/JAK/STAT3 signaling drives metastasis and therapy resistance, NGO platforms have been applied to silence STAT3 via nucleic acid delivery and to reduce tumor bioenergetics and invasiveness [42]. While these contexts differ from autoimmunity or chronic inflammatory skin disease, they converge mechanistically on NGO dampening STAT3-driven programs, reinforcing our proposed mtROS–STAT3 linkage in psoriatic T cells.

NGO quantum dots have also been shown to ameliorate inflammatory colitis by decreasing IL-6 levels, restraining Th17 responses, shifting macrophages toward an anti-inflammatory M2 phenotype, and increasing Tregs [43]. These immunological effects were accompanied by improvements in weight, histology, and disease activity indices in mouse models. As inflammatory colitis is a well-recognized comorbidity of psoriasis and both diseases are driven by the IL-23/Th17 axis [44, 45], these data support the possible beneficial effect of NGO on psoriatic inflammation.

Psoriasis is sustained by the IL-23/IL-17 axis and a skin-tropic TRM cell reservoir. In particular, local IL-23 drives in situ proliferation and retention of TRM17 cells, while keratinocyte–IL-17 feedforward circuits amplify inflammation [46, 47]. As IL-23 engages IL-23R to drive TYK2–STAT3 signaling, and NGO decreased STAT3 activation in our study, NGO may provide therapeutic benefit in psoriasis. In addition to simply blocking the IL-23/IL-17 pathway, NGO also improves mitochondrial dysfunction and ROS stress, which are important contributors to disease [48, 49], implying the potential for improved efficacy over existing agents. Notably, anti-IL-23 monoclonal antibodies often produce only a transient reduction in TRM cells, and about 90% of patients relapse within 2 years after discontinuation [12, 50, 51]. By acting on mechanisms beyond the IL-23/IL-17 axis, NGO may help to sustain psoriasis remission for longer periods.

Our study had a number of limitations. First, in vivo efficacy was demonstrated in an acute IMQ-induced model with short-term exposure, and human data were obtained from PBMCs rather than lesional skin. Durability and TRM cell reprogramming will require further studies in chronic/relapse models. We also tested a single intraperitoneally delivered NGO formulation, so topical/intradermal delivery, material standardization, dermal pharmacokinetics, and toxicology remain to be defined. Despite these constraints, the concordant metabolic, signaling, and cellular readouts across in vitro, mouse model, and patient systems provide strong proof-of-concept for NGO as a TRM cell-modulating strategy in psoriasis.

In conclusion, our findings establish NGO as a metabolic signaling modulator that can restrain mtROS and STAT3 activity, thereby attenuating pathogenic TRM cells in psoriasis. Furthermore, NGO also significantly reduces proinflammatory CD4^+^/CD8^+^ T cells and key cytokines such as IL-23/IL-17, while restoring regulatory T cells, indicating comprehensive immune regulation related to psoriasis pathogenesis. Moreover, mtROS suppression and metabolic recovery in total lymphocytes both in mice and psoriasis patients also highlight NGO’s systemic effect on immune cell metabolism beyond just TRM໿ cells. Based on these findings, NGO has potential for more durable disease control than current biologics in psoriasis.

## Conclusion

This study demonstrated that NGO can provide potent therapeutic benefits in psoriasis by restoring mitochondrial homeostasis and suppressing not only IL-23/IL-17 secretion level, IL-17^+^CD4^+^ cells but also pSTAT3–IL-17–expressing CD8^+^ TRM cells. NGO treatment effectively reduced pathogenic TRM cell populations, alleviated skin inflammation and hyperplasia, and increased Tregs’ activity in both the mouse model and patient-derived immune cells. Mechanistically, NGO couples mitochondrial modulation with immune regulation to disrupt core drivers of disease persistence. These results establish NGO as a promising immunometabolic strategy for durable intervention in chronic inflammatory skin disease by modulating the metabolism of pathogenic CD8TRM and the comprehensive immune cell populations associated with psoriasis pathogenesis.

## Supplementary Information

Supplementary Material 1

Supplementary Material 2

Supplementary Material 3

## Data Availability

This study did not involve the generation or analysis of any data sets.

## Declarations

### **Author information**

Tae Ho Kim, Chae Rim Lee, Kyoung Min Choi, Soon Won Choi, Seon-Yeong Lee, A Ram Lee, Jaechul Ryu, Jung Won Choi, Young Mi Moon, Joonhyuck Park, Haeyoun Choi contributed equally to this work.
