# Supplementary figures and images for: NGO ameliorates psoriasis by modulating mitochondrial function and suppressing pSTAT3–IL-17–expressing CD8+ TRM cells
Source: J Nanobiotechnology. 2026 Jan 16;24:133. doi: 10.1186/s12951-025-04020-7 (PMC12879470; doi:10.1186/s12951-025-04020-7)

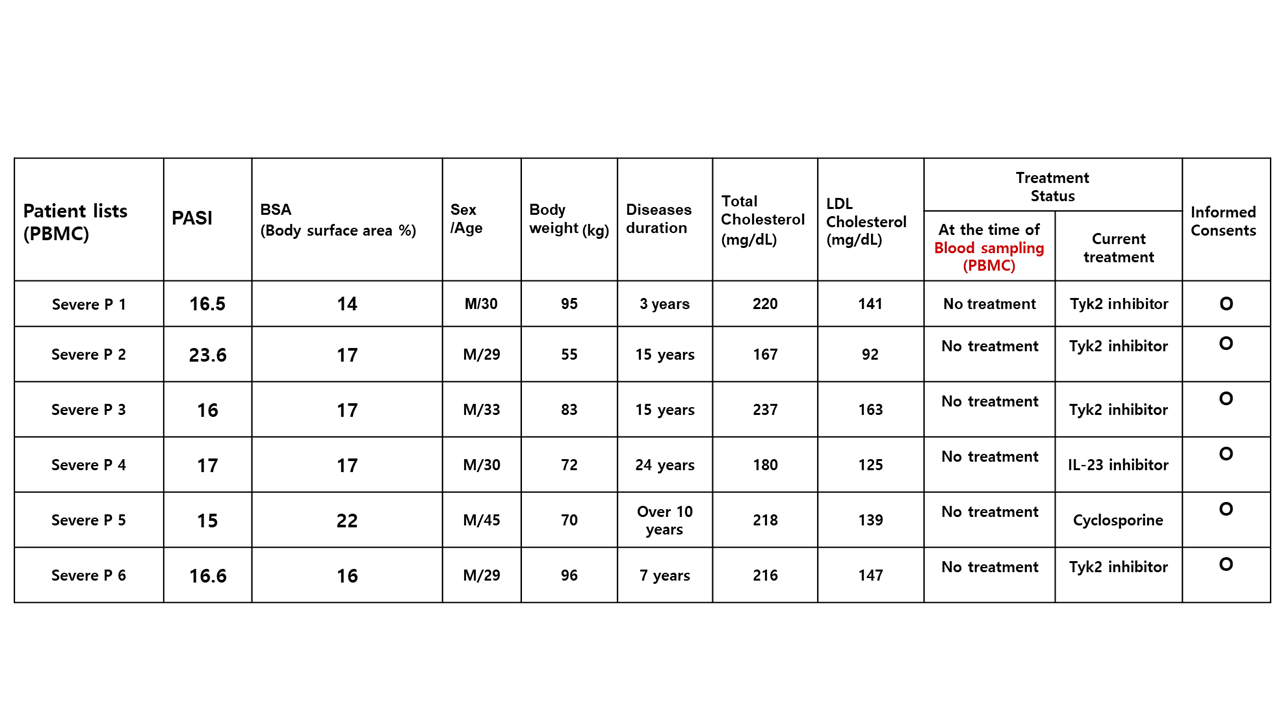

Supplement: Supplementary file 1 — Supplementary Material 1 [file 12951_2025_4020_MOESM1_ESM.tif]
