# Supplementary material for: NGO ameliorates psoriasis by modulating mitochondrial function and suppressing pSTAT3–IL-17–expressing CD8+ TRM cells
Source: J Nanobiotechnology. 2026 Jan 16;24:133. doi: 10.1186/s12951-025-04020-7 (PMC12879470; doi:10.1186/s12951-025-04020-7)
Supplement: Supplementary file 2 — Supplementary Material 2 [file 12951_2025_4020_MOESM2_ESM.docx]

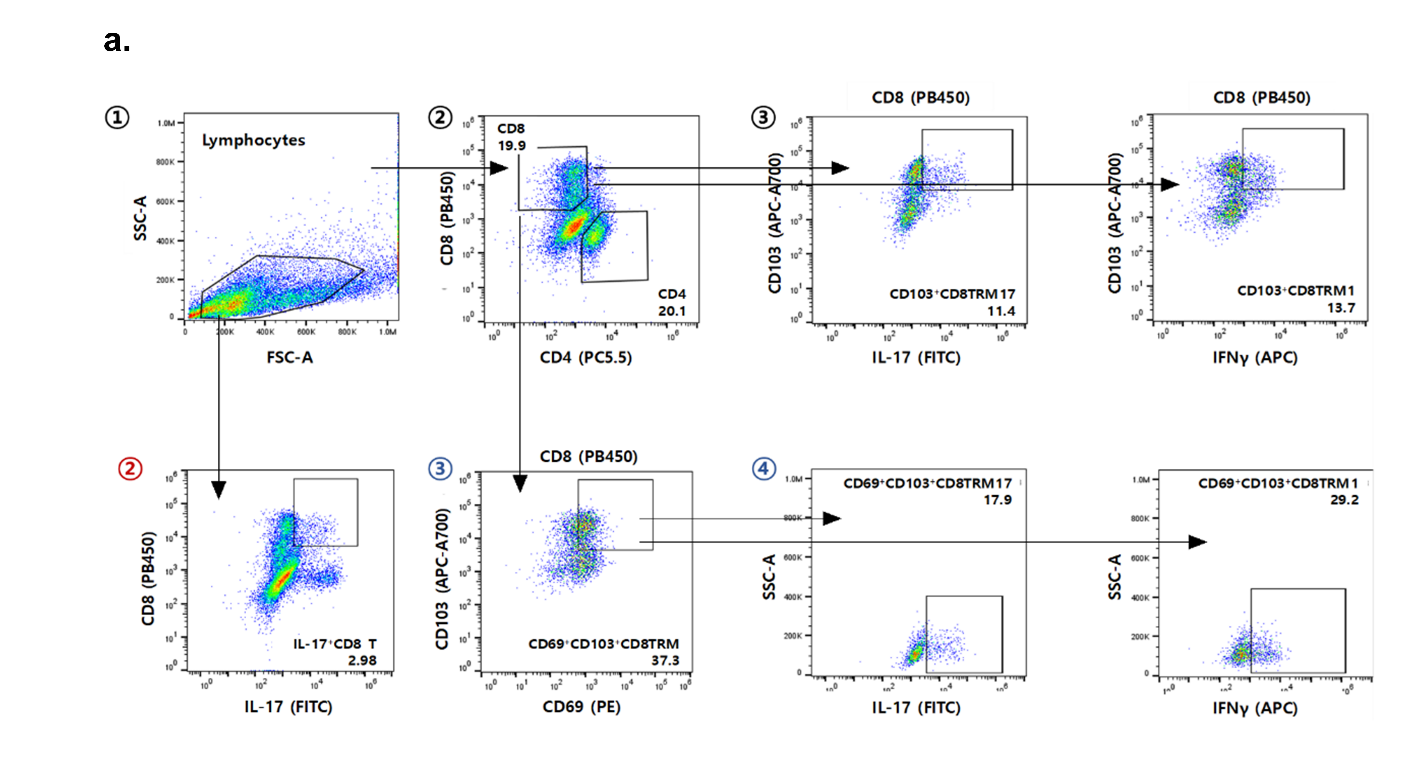
**∙ Supplementary Figure**

**Supplementary Figure 1. Flow cytometric gating strategy for identification of IL-17–producing CD8^+^ T cell subsets including tissue-resident memory populations in mouse splenocytes.**

Gating strategy for identification of IL-17–producing CD8^+^ T cell subsets including tissue-resident memory populations in mouse splenocytes. Lymphocytes were gated based on FSC-A/SSC-A, followed by CD8^+^ and CD4^+^ T cell separation. IL-17–producing CD8^+^ T cells were quantified by intracellular cytokine staining to assess frequency of CD8^+^IL-17^+^double positive cells, directly from the lymphocyte gate. For epidermal TRM like subset analysis, CD8^+^ T cells co-expressing CD103 and IL-17 or IFN-γ (frequency of CD103^+^IL-17^+^ or CD103^+^ IFN-γ^+^ in CD8^+^ T cells) were identified. Double-positive TRM phenotype was determined by gating for simultaneous CD69 and CD103 expression in CD8^+^ T cells (frequency of CD69^+^CD103^+^ in CD8 T cells) with IL-17 or IFN-γ production (frequency of IL-17^+^ or IFN-γ^+^ cells in CD69^+^CD103^+^CD8^+^ T cells) measured within this population. The full gating strategy is detailed in the schematic.


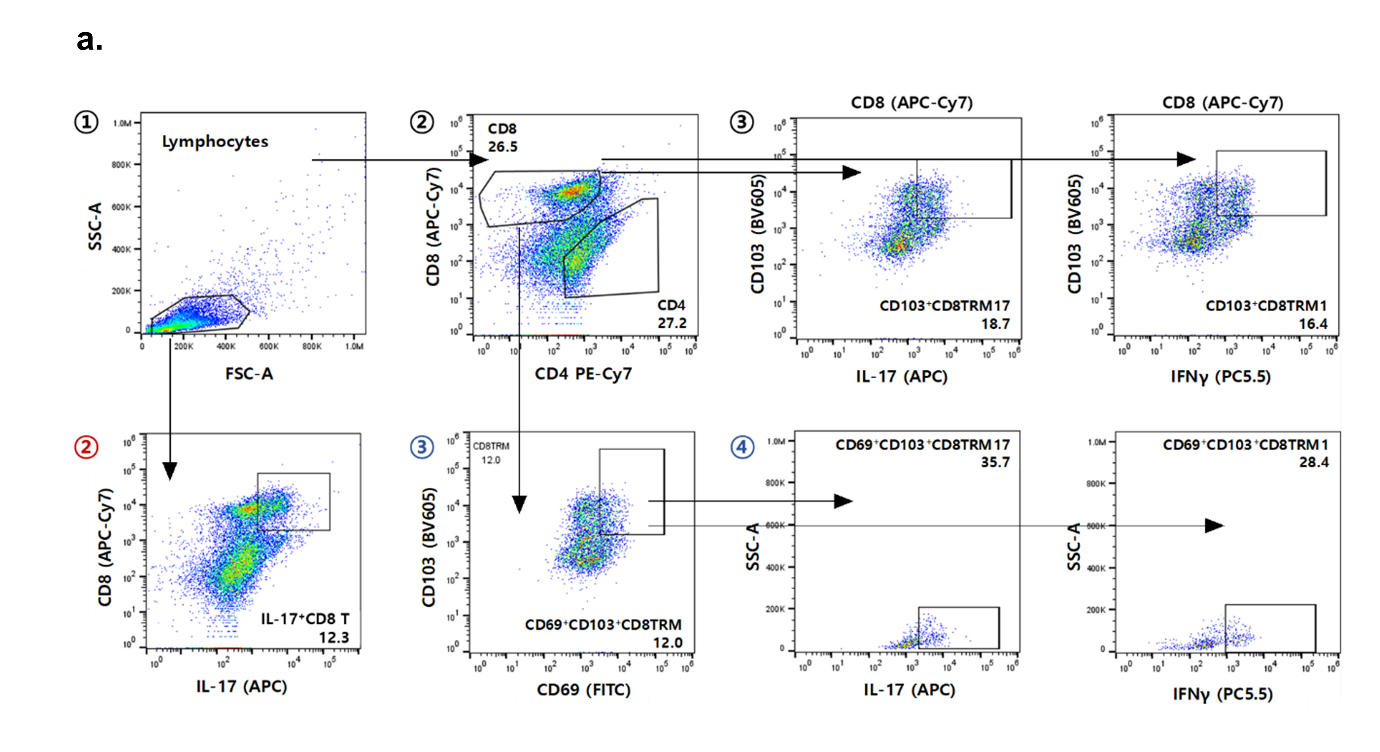
**Supplementary Figure 2. Flow cytometric gating strategy for identification of IL-17–producing CD8^+^ T cell subsets, including tissue-resident memory populations, in PBMCs from psoriasis patients.**

Gating strategy for delineation of IL-17–producing CD8^+^ T cell subsets, including tissue-resident memory subsets in peripheral blood mononuclear cells (PBMCs) from psoriasis patients. Lymphocytes were identified by FSC-A/SSC-A profiles prior to segregation of CD8^+^ and CD4^+^ T cells. Frequencies of IL-17–producing CD8^+^ T cells were determined via intracellular cytokine staining, enabling direct detection of CD8^+^IL-17^+^ double positive events from the initial lymphocyte population. For the assessment of epidermal TRM like cells, CD8^+^ T cells co-expressing CD103 and IL-17 or IFN-γ (frequency of CD103^+^IL-17^+^ or CD103^+^IFN-γ^+^ in CD8^+^ T cells) were analyzed. Dual-marker TRM subsets were further gated based on co-expression of CD69 and CD103 in CD8^+^ T cells (frequency of CD69^+^CD103^+^ in CD8^+^ T cells), followed by measurement of IL-17 or IFN-γ production (frequency of IL-17^+^ or IFN-γ^+^ cells in CD69^+^CD103^+^CD8^+^ T cells) within this compartment. The full stepwise gating workflow is illustrated in the schematic.

**∙ Supplementary Table**


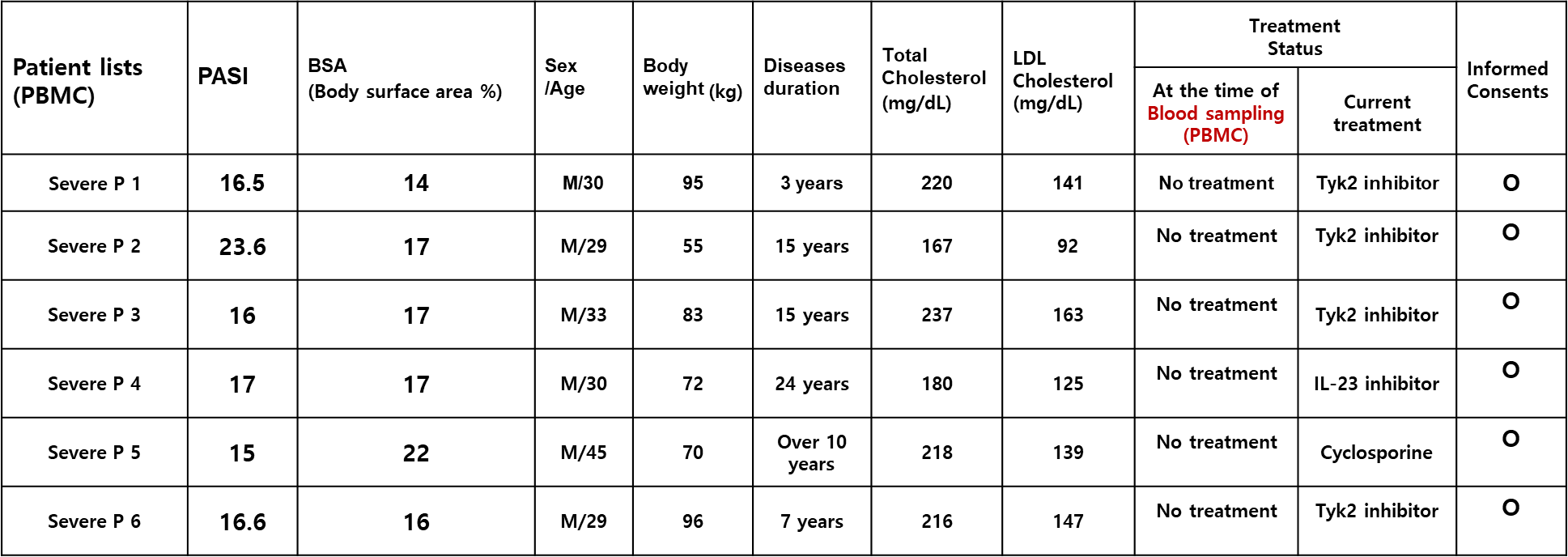


**Clinical information of psoriasis patients:** Clinical information and treatment status of 6 severe psoriasis patients included for peripheral blood mononuclear cell (PBMC) analysis. Patient data includes Psoriasis Area and Severity Index (PASI), body surface area (BSA, %), sex and age, body weight, disease duration, total cholesterol, low-density lipoprotein (LDL) cholesterol, therapeutic regimen at the time of blood sampling, as well as current treatment and informed consent status.
