# Supplementary material for: NGO ameliorates psoriasis by modulating mitochondrial function and suppressing pSTAT3–IL-17–expressing CD8+ TRM cells
Source: J Nanobiotechnology. 2026 Jan 16;24:133. doi: 10.1186/s12951-025-04020-7 (PMC12879470; doi:10.1186/s12951-025-04020-7)
Supplement: Supplementary file 3 — Supplementary Material 3 [file 12951_2025_4020_MOESM3_ESM.pdf]

Western Blot Gel Full image

Kim et al.

Figure 2d.

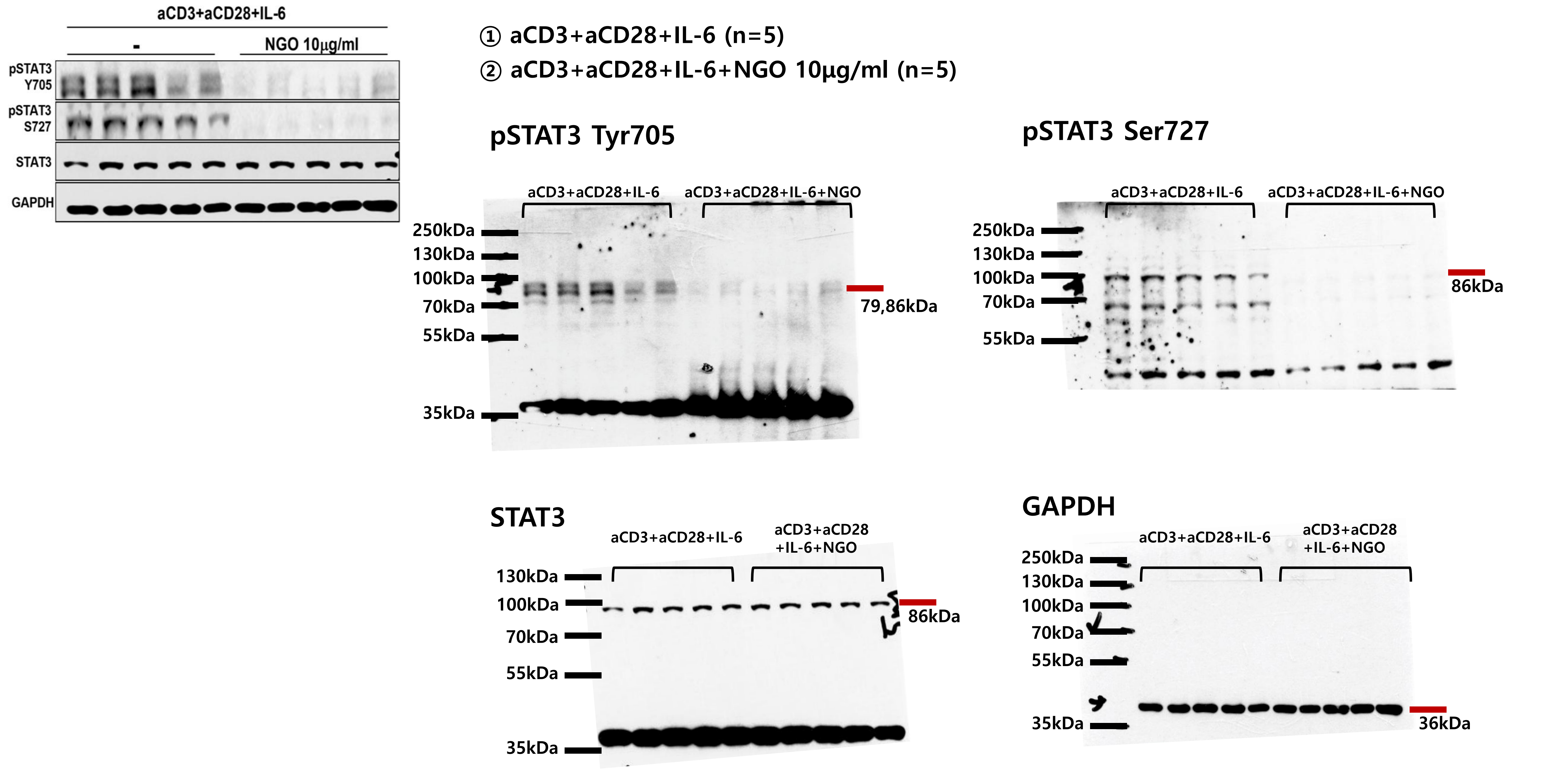
aCD3+aCD28+IL-6
-
NGO 10μg/ml
pSTAT3 Y705
pSTAT3 S727
STAT3
GAPDH
① aCD3+aCD28+IL-6 (n=5)
② aCD3+aCD28+IL-6+NGO 10μg/ml (n=5)
pSTAT3 Tyr705
aCD3+aCD28+IL-6
aCD3+aCD28+IL-6+NGO
250kDa
130kDa
100kDa
70kDa
55kDa
35kDa
79,86kDa
pSTAT3 Ser727
aCD3+aCD28+IL-6
aCD3+aCD28+IL-6+NGO
250kDa
130kDa
100kDa
70kDa
55kDa
86kDa
STAT3
aCD3+aCD28+IL-6
aCD3+aCD28
+IL-6+NGO
130kDa
100kDa
70kDa
55kDa
35kDa
86kDa
GAPDH
aCD3+aCD28+IL-6
aCD3+aCD28
+IL-6+NGO
250kDa
130kDa
100kDa
70kDa
55kDa
35kDa
36kDa

Figure 4a.

① SKG+IMQ (n=6)
② SKG+IMQ+NGO 6.7mpk (n=6)

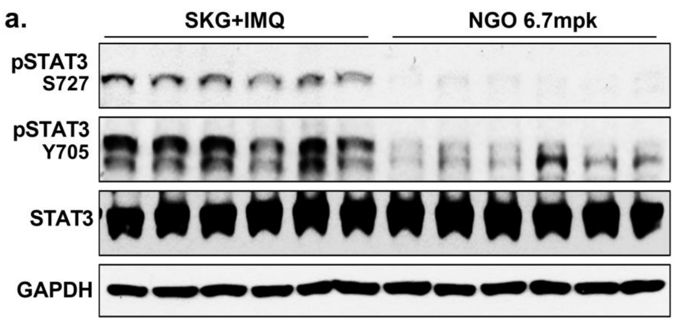

pSTAT3 Ser727

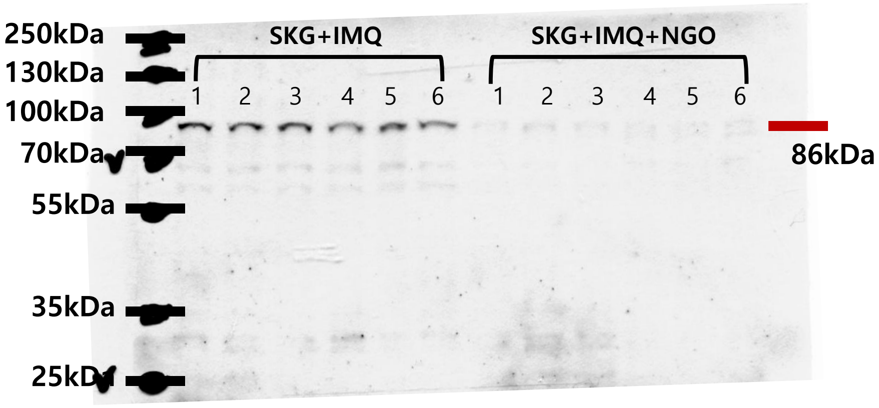

pSTAT3 Tyr705

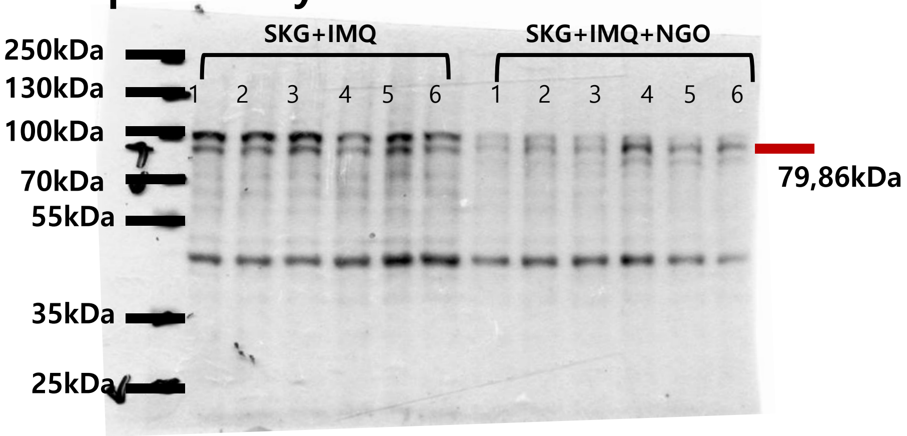

STAT3

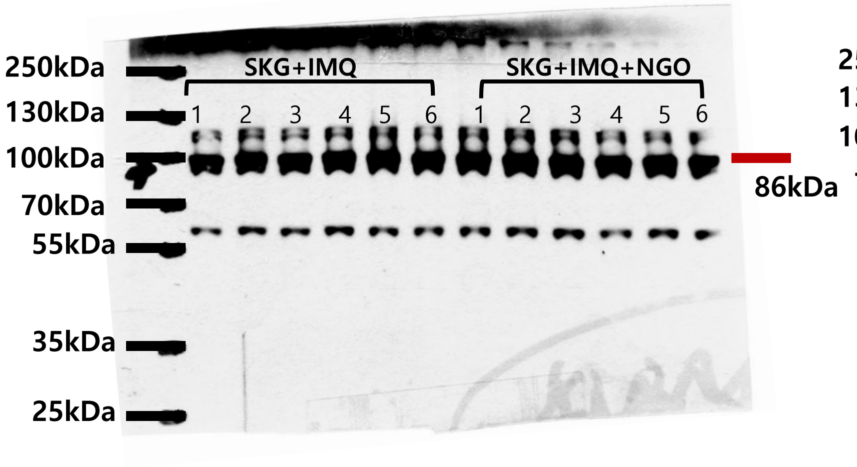

GAPDH

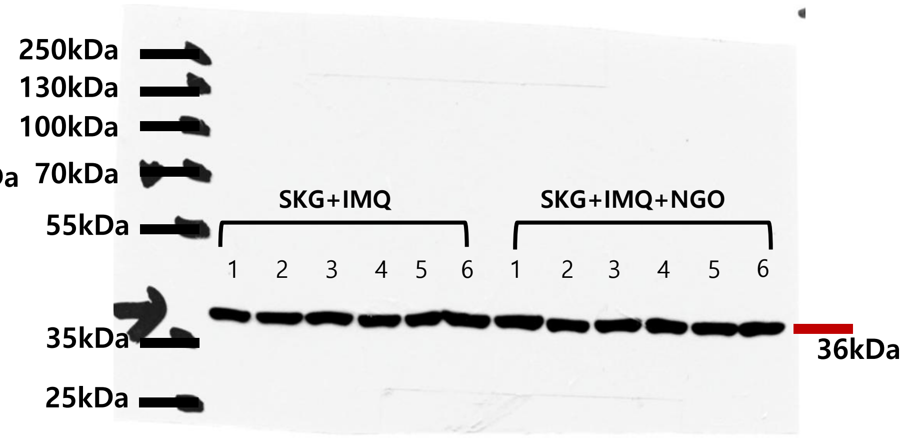

Figure 5e.

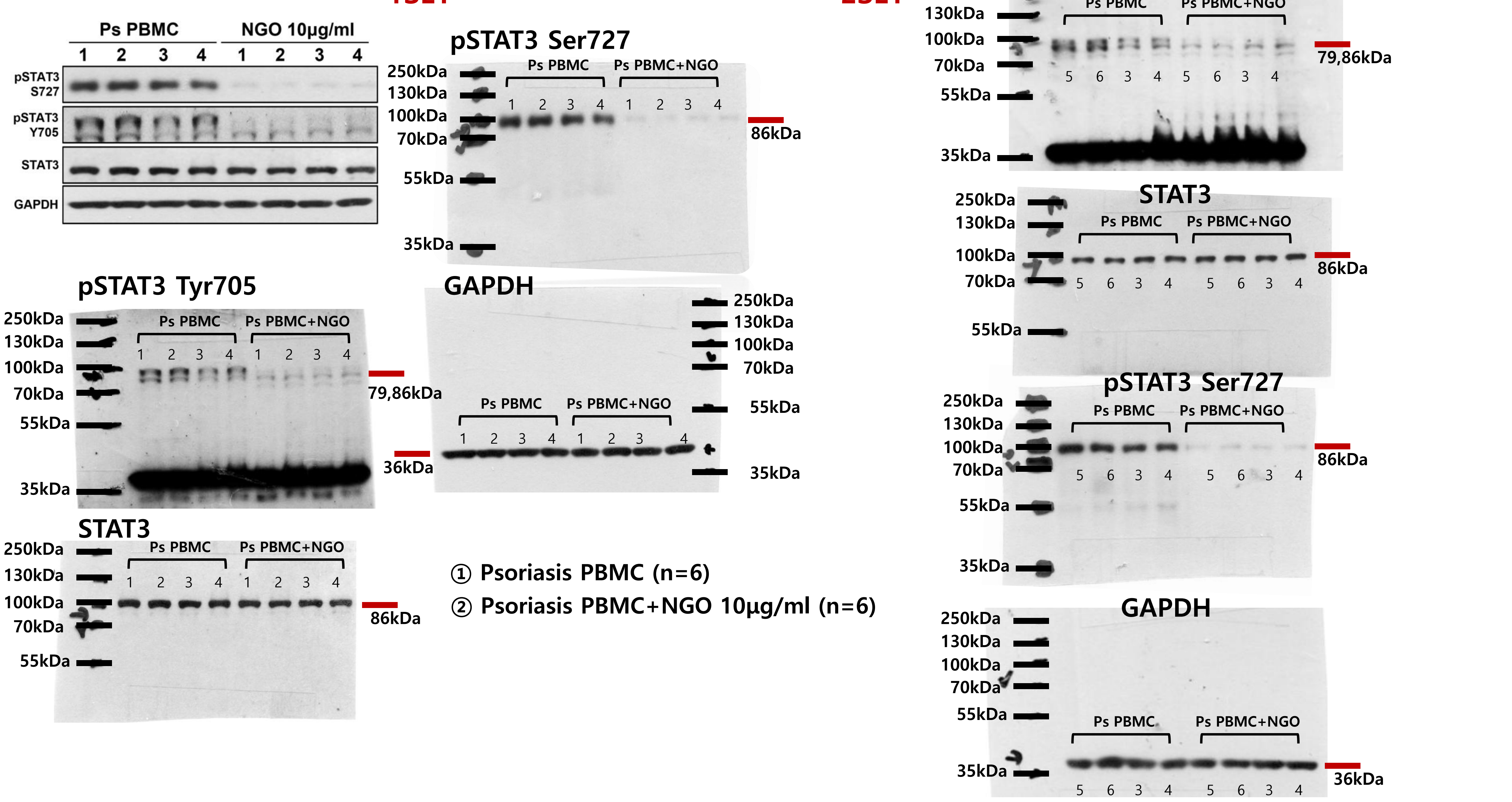
1SET
2SET
Ps PBMC
NGO 10μg/ml
1 2 3 4 1 2 3 4
pSTAT3 S727
pSTAT3 Y705
STAT3
GAPDH
pSTAT3 Ser727
250kDa
130kDa
100kDa
70kDa
55kDa
35kDa
Ps PBMC
Ps PBMC+NGO
86kDa
pSTAT3 Tyr705
79,86kDa
GAPDH
36kDa
STAT3
5 6 3 4 5 6 3 4
① Psoriasis PBMC (n=6)
② Psoriasis PBMC+NGO 10μg/ml (n=6)
